# Effect of Carbon Sources on the Production of Volatile Organic Compounds by *Fusarium verticillioides*

**DOI:** 10.3390/jof8020158

**Published:** 2022-02-05

**Authors:** Fernanda Achimón, Vanessa D. Brito, Romina P. Pizzolitto, Julio A. Zygadlo

**Affiliations:** 1 Multidisciplinary Institute of Plant Biology (IMBIV-CONICET), National University of Cordoba, Cordoba X5016GCA, Argentina; fachimon@imbiv.unc.edu.ar (F.A.); vbrito@imbiv.unc.edu.ar (V.D.B.); juliozyg29@gmail.com (J.A.Z.); 2 Science and Food Technology Institute (ICTA), National University of Cordoba, Cordoba X5016GCA, Argentina; 3 Chemistry Department, Faculty of Exact, Physical and Natural Science, National University of Cordoba, Cordoba X5016GCA, Argentina

**Keywords:** *Fusarium verticillioides*, volatile organic compounds, mycodiesel, carbon sources

## Abstract

The aim of the present study was to evaluate the effect of different carbon sources on the hydrocarbon-like volatile organic compounds (VOCs) of *Fusarium verticillioides* strain 7600 through a Principal Component Analysis approach, and to explore their diesel potential by using data from the literature. The fungus was cultivated in GYAM culture medium, and five carbon sources were evaluated: glucose, sucrose, xylose, lactose, and fructose. The VOCs were collected using a close-loop apparatus and identified through GC-MS. The same profile of 81 VOCs was detected with all treatments, but with different relative percentages among carbon sources. The production of branched-chain alkanes (30 compounds) ranged from 25.80% to 38.64%, straight-chain alkanes (12 compounds) from 22.04% to 24.18%, benzene derivatives (12 compounds) from 7.48% to 35.58%, and the biosynthesis of branched-chain alcohols (11 compounds) was from 6.82% to 16.71%, with lower values for the remaining groups of VOCs. Our results show that *F. verticillioides* has the metabolic potential to synthesize diesel-like VOCs. Further research should include the optimization of culture conditions other than carbon sources to increase the production of certain groups of VOCs.

## 1. Introduction

Volatile Organic Compounds (VOCs) are carbon-based compounds of low molecular mass that enter the gas phase at normal temperatures and pressure. Over 300 VOCs have been described for fungi, including hydrocarbons, aldehydes, ketones, alcohols, phenols, thioesters, and benzene derivatives, which are synthesized during both primary and secondary metabolism [1,2]. Several biotechnological applications have been reported for fungal VOCs, such as biocontrol agents, food additives, perfumery, and biofuels [3,4,5]. In this context, VOCs have certain properties that are consistent with those of good diesel fuels, such as relatively low molecular weight, high cetane number, and high energy content [6]. The biosynthesis of straight-chain and branched-chain hydrocarbons (alkanes and alkenes), cyclic alkanes, benzene derivatives, alcohols, ketones, esters, and terpenes has been reported for different endophytic fungal species grown in cellulose-based and synthetic culture media [7,8,9,10,11].

Filamentous fungi offer many advantages that make them suitable organisms for VOC production. Notably, they grow fast and have short life cycles, they are not influenced by external factors (such as climate or soil conditions), they are amenable to genetic improvements, and they can be easily scaled up from laboratory to bioreactor level to achieve mass production [12]. However, the ability of a certain fungal species to grow and produce the VOCs of interest will ultimately depend on the culture composition and cultivation conditions, e.g., carbon and nitrogen sources, pH and temperature, age of culture, etc. [13]. Different aspects of fungal metabolism, such as the hyphal branching pattern, the growth rate and sporulation, the accumulation of lipids and organic acids, and the production of pigments and toxins were markedly different according to the carbon source provided in the culture medium in different fungal species [4,14]. However, despite the immense biotechnological potential of fungal VOCs [5], the optimization of their production by using different carbon sources has barely been addressed in the literature.

*Fusarium verticillioides* is a phytopathogen of maize and the major causal agent of stalk and ear rot worldwide. Moreover, *F. verticillioides* also exists as a symptomless intercellular endophyte [15]. A previous study evaluated the production of ethanol using an endophytic strain of *F. verticillioides* grown with different carbon sources as substrates [16]. However, ethanol is not the most desirable fuel because of its low energy content and high hygroscopicity, which can lead to engine fouling and transportation problems [3]. On the other hand, *F. verticillioides* has been recognized as an oleaginous filamentous fungus, since it can accumulate high amounts of fatty acids in its cells [17]. Hydrocarbons are organic compounds consisting entirely of carbon and hydrogen, and comprise saturated, unsaturated, branched, aromatic, and cyclic molecular structures [18]. Additionally, these VOCs can be implicated in other reactions to form different oxygenated compounds, such as alcohols, esters, acids, aldehydes, and ketones, among others. It is well-known that the biosynthesis of VOCs by filamentous fungi proceeds through different primary and secondary metabolic pathways [1], where the metabolism of fatty acids plays a prevalent role. A handful of VOCs derived from lipid peroxidation (i.e., 3-octanol, 3-octanone, 1-octen-3-ol, and 1-octen-3-one) and the terpene profile of *F. verticillioides* have been evaluated under different culture conditions [4,19,20,21,22]; however, most of the hydrocarbon-derived VOCs emitted by *F. verticillioides* remain unexplored.

Accordingly, the aims of the present work were: (i) to study the hydrocarbons and hydrocarbon-derived VOCs produced by *F. verticillioides* strain 7600; (ii) to evaluate the effect of different carbon sources on the VOC profile of *F. verticillioides* strain 7600 using Principal Component Analysis (PCA); and (iii) to explore the mycodiesel potential of these VOCs using data from the literature.

## 2. Materials and Methods

### 2.1. Fungal Strain and Inoculum Preparation 

*Fusarium verticillioides* strain 7600 (or M3125) was originally supplied by Dr. Robert Proctor, United States Department of Agriculture, Agricultural Research Service, National Center for Agricultural Utilization Research, Peoria, IL, United States. This fungal strain has been genomically characterized (Genbank Accession AAIM00000000.2; PRJNA15553).

The inoculum of *F. verticillioides* was prepared from sporulated fungal cultures developed on Potate Dextrose Agar (PDA; Britania; Buenos Aires, Argentina) for 7 days at 28 °C. Spores were collected by adding 5 mL of sterile distilled water and shaking by hand. Spore concentration was adjusted to 1 × 10^6^ spores/mL using an Improved Neubauer chamber (Marienfeld; Lauda-Königshofen; Germany) [23].

### 2.2. Cultivation on Different Carbon Sources

*Fusarium verticillioides* was cultivated in 100 mL-flaks containing 40 mL of Glucose Yeast Asparagine Malic acid liquid synthetic medium (GYAM) and inoculated with 500 µL of the spore suspension (1 × 10^6^ spores/mL). GYAM is a liquid synthetic medium characterized by a high content of C, which is important to promote fatty acid metabolism [24]. GYAM culture medium was prepared with 0.67 g of malic acid, 1.20 g of 1-asparagine, 0.1 g of NaCl, 0.77 g of K_2_HPO_4_, 0.49 g of MgSO_4_, 0.98 g of CaCl_2_, 0.50 g of yeast extract and 40 g of a carbon source consisting of either glucose, fructose, xylose, sucrose, or lactose, per liter. The pH of the culture medium was adjusted to 3 with H_3_PO_4_. The flasks were incubated in a rotary shaker for 5 days at 25 °C in the dark. On the 5th day of incubation, a sampling system of VOCs was installed. For mycelium dry weight (DW) determination, cultures were centrifuged at 5000 rpm for 10 min, and the pellets were dried at 60 °C until constant weight. Uninoculated sterile culture media were used as control samples, and the experiments were repeated twice in quintuplicate.

### 2.3. Collection and Identification of VOCs

The collection of VOCs was performed from the 5th to the 7th day of incubation, as previous studies detected maximum VOC production in this time frame with other species of *Fusarium* [25]. A closed-loop system, as described by Achimón et al. [21], was used. Briefly, each 100-mL flask was closed with a rubber stopper with a gas inlet and a gas outlet coupled to a trap. The trap consisted of a glass tube (5 mm diameter) filled with 400 mg of Super-Q adsorbent (mesh size 80/100; Alltech Associates; Chicago, IL, USA). To eliminate impurities, the air first passed through a trap filled with sterile cotton and then through a trap filled with 10 g of activated charcoal before flowing into the fungal culture [22]. For the elution of VOCs from the Super-Q traps, a mixture of pentane-ethyl ether (1:1 *v*/*v*) was used. The eluate was concentrated to 100 µL and 1 µL was injected into a Perkin Elmer SQ8 Gas Chromatographer/Mass Spectrometer (GC/MS; PerkinElmer; Waltham, MA, USA). The injection port was set at 250 °C; the temperature of the DB5 column (60 m × 0.25 mm × 0.25 μm; Elite 5 MS PerkinElmer; Waltham, MA, USA) was kept at 40 °C for 3 min, increased to 240 °C at a rate of 4 °C/min, and then kept constant for 10 min. Helium was used as the carrier gas, and the flow rate was 1 mL/min. Electron ionization (EI) on the Mass Spectrometer was set at 70 eV with a mass scan range of *m/z* 40–300 atomic mass units (amu). Kovats retention indices (KI) were calculated after an analysis of C8–C21 alkane series. Volatile compounds were identified by matching their mass spectrum and KI with those from the library of the National Institute of Standards and Technology (NIST14). The amount of each volatile compound was expressed as a relative percent by peak area normalization.

### 2.4. Statistical Analysis

After identification, fungal VOCs were classified into the following categories: straight-chain alkanes, branched-chain alkanes, cyclic alkanes, alkenes, benzene derivatives, straight-chain alcohols, branched-chain alcohols, esters, and ketones and aldehydes. Significant differences on the production of each group of VOCs among carbon sources were analyzed by a one-way ANOVA (*p* < 0.05) followed by a DGC test. In addition, we performed a Principal Component Analysis (PCA) within each group of VOCs to better understand the effect of different carbon sources on VOCs biosynthesis by *F. verticillioides*. All statistical analyses were conducted using the Infostat Software 2020 Professional (National University of Cordoba).

## 3. Results

### VOCs Production by F. verticillioides Strain 7600

All carbon sources supported the growth of *F. verticillioides* 7600. The fungal biomass (expressed as mg of DW per ml of culture medium) was significantly higher in cultures supplemented with sucrose (6.81 ± 0.13 mg DW/mL), glucose (6.35 ± 0.13 mg DW/mL), and fructose (5.98 ± 0.48 mg DW/mL). Statistically significant lower values of fungal biomass were detected with xylose (4.34 ± 0.11 mg DW/mL) and lactose (3.79 ± 0.19 mg DW/mL) as carbon sources. 

Regarding the volatile profile, a total of 81 VOCs were identified and classified as straight-chain alkanes, branched-chain alkanes, cyclic alkanes, alkenes, benzene derivatives, straight-chain alcohols, branched-chain alcohols, esters, or ketones and aldehydes. The list of the VOCs produced is shown in Table 1, along with their relative percentage and Kovats retention indices (KI). All carbon sources supported the production of the same volatile profile by *F. verticillioides* strain 7600. The most represented group was branched-chain alkanes (30 compounds), followed by straight-chain alkanes and benzene derivatives (12 compounds each), and branched-chain alcohols (11 compounds). A wide range of other compounds were also produced in lower amounts, including esters (5 compounds), cyclic alkanes (4 compounds), alkenes (2 compounds), and ketones and aldehydes (3 compounds). However, regardless of the number of compounds identified within each group, the total percentage of VOCs was similar among different groups of VOCs and carbon sources. Such was the case of glucose and lactose, that showed similar percentages of straight-chain and branched-chain alkanes. Likewise, sucrose presented similar amounts of straight-chain alkanes and benzene derivatives (Table 1). On the other hand, even though the number of detected compounds was 12 for both straight-chain alkanes and benzene derivatives, the latter were produced in higher amounts in fungal cultures with glucose and lactose.

**Figure 1:**
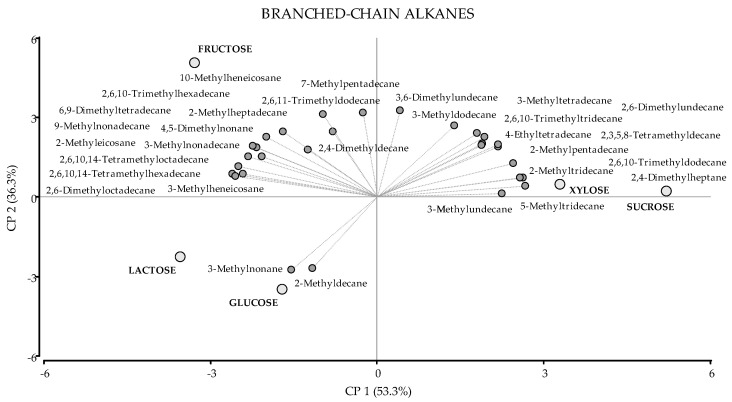
PCA of Branched-chain alkanes. A total of 35 variables were included: 30 VOCs (dark grey circles) and 5 carbon sources (light grey circles). The first two principal components accounted for 53.3% and 36.3% of the total variation, respectively.

Branched-chain alkanes ranged from 9 to 22 carbon atoms. The majority had only one methyl-substitution (15 compounds); seven compounds had two, and seven had more than two methyl-substitutions (see Table 1). Some of the branched-chain alkanes detected in higher amounts in the VOC mixture of this fungus were: 4.5-dimethylnonane, 3-methyldodecane, 2,6,11-trimethyldodecane, 2-methyltridecane, 3-methyltetradecane, 7-methylpentadecane, and 2-methylpentadecane. The production of branched-chain alkanes was statistically higher in fungal cultures with fructose (38.64%), sucrose (37.60%), and xylose (35.56%). However, the different compounds were present in higher amounts with different carbon sources, as evidenced in the PCA (see Figure 1). The two principal components (PCA1 and PCA2) of this analysis accounted for 89.6% of the total variance (Figure 1). In a PCA, the orientation of vectors as well as the angles between them reflect the correlation among variables, in this case, VOCs and carbon sources. For example, 3-methylnonane and 2-methyldecane were located close to glucose and lactose, the carbon sources where these compounds were present in higher amounts (Table 1). However, most branched-chain alkanes were positioned close to sucrose, xylose, and fructose (Figure 1), indicating that they were detected in higher amounts in fungal cultures supplemented with those carbon sources (Table 1). In addition, as shown in the biplot, fungal cultures with sucrose and xylose showed a similar pattern of VOCs, which is consistent with the data in Table 1. In fact, most compounds that were present in the highest amounts in fungal cultures with sucrose were followed by xylose, and vice versa. For example, xylose was the second carbon source after sucrose, where the fungus produced 2,3,5,8-tetramethyldecane, 5- and 2-methyltridecane, 2,6,10-trimethyldodecane, and 2,6,10-trimethyltridecane. Likewise, sucrose was the second carbon source after xylose, where the production of 3-methyltetradecane, 4-ethyltetradecane, and 2-methylpentadecane was enhanced. Moreover, methyl-substituted hexadecanes, heptadecanes, octadecanes, nonadecanes, eicosanes, and heneicosanes were present in higher amounts in fungal cultures with fructose, which is in agreement with the results in Table 1.

**Figure 2:**
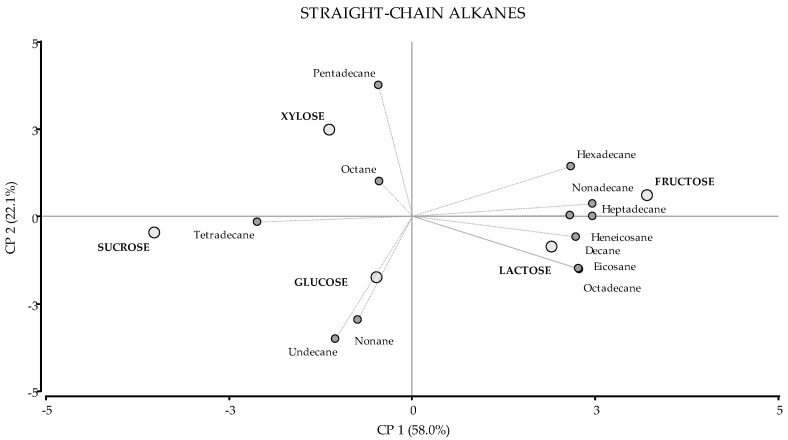
PCA of Straight-chain alkanes. A total of 17 variables were included: 12 VOCs (dark grey circles) and 5 carbon sources (light grey circles). The first two principal components accounted for 58.0% and 22.1% of the total variation, respectively.

Straight-chain alkanes were less represented in the analyses, with only 12 compounds detected. Even though the relative percentages of certain compounds were rather different according to the carbon source provided, there were no statistically significant differences in the total production of straight-chain alkanes among the various carbon sources (Table 1). The two principal components accounted for 80.1% of the differentiation (Figure 2). As shown in the biplot, lactose and fructose were plotted close to each other and segregated from the remaining carbon sources by the PCA1, because higher amounts of decane, hexadecane, heptadecane, octadecane, nonadecane, eicosane, and heneicosane were achieved with these carbon sources (Table 1).

**Figure 3:**
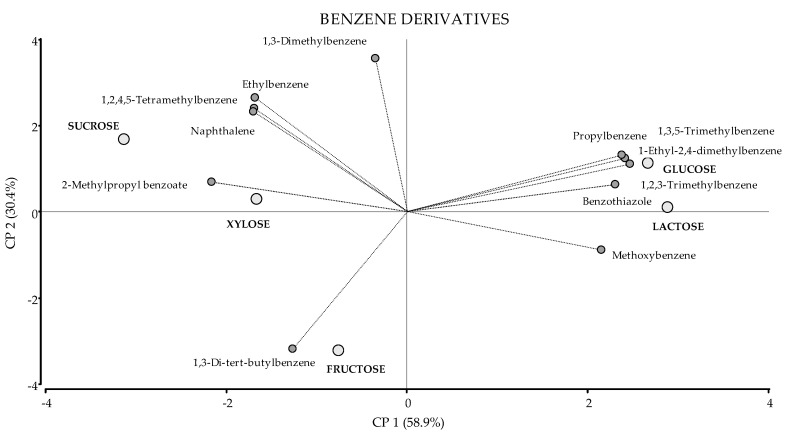
PCA of Benzene derivatives. A total of 17 variables were included: 12 VOCs (dark grey circles) and 5 carbon sources (light grey circles). The first two principal components accounted for 58.9% and 30.4% of the total variation, respectively.

Regarding benzene derivatives, the greatest amounts were detected in fungal cultures supplemented with glucose and lactose, i.e., 35.58% and 32.33%, respectively. The production of these VOCs was significantly lower in the remaining carbon sources, particularly in fructose, where the relative percentage was almost five times lower (Table 1). The PCA1 and PCA2 of this analysis accounted for 89.3% of the total variance (Figure 3). Glucose and lactose showed high amounts of the same compounds: propylbenzene, 1,3,5-trimethylbenzene, 1,2,3-trimethylbenzene, 1-ethyl-2,4-dimethylbenzene, benzothiazole, and methoxybenzene, explaining their position in the score plot (Figure 3). Furthermore, ethylbenzene, 1,3-dimethylbenzene, 1,2,4,5-tetramethylbenzene, and naphthalene were produced in higher percent in fungal cultures with sucrose while 1,3-di-tert-butylbenzene and 2-methylpropyl benzoate were higher with fructose and xylose, respectively (Figure 3).

**Figure 4:**
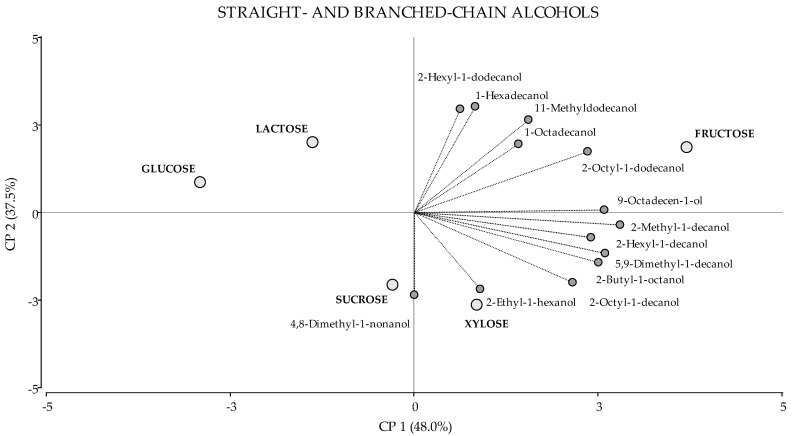
PCA of Straight- and branched-chain alcohols. A total of 18 variables were included: 13 VOCs (dark grey circles) and 5 carbon sources (light grey circles). The first two principal components accounted for 48.0% and 37.5% of the total variation, respectively.

Straight- and branched-chain alcohols are represented in the same biplot (Figure 4). In this analysis, the PCA1 and PCA2 accounted for 85.5% of the total variance. Fructose was the carbon source where the total production of branched-chain alcohols was highest (with statistical significance) compared to the remaining carbon sources. Regarding straight-chain alcohols, both fructose and lactose showed the greatest production (Table 1). Indeed, most of the detected alcohol compounds were present in higher amounts in fungal cultures with fructose (Table 1 and Figure 4). In addition, none of the alcohol compounds increased in abundance in the presence of glucose or lactose, which explains their segregation in the score plot (Figure 3).

Other groups of VOCs were detected in lower amounts in the experiments, such as esters, cyclic alkanes, alkenes, and ketones and aldehydes. Regarding esters, most of them were present in higher amounts in fungal cultures with fructose. However, there were no significant differences in the total ester production between fructose and lactose (Table 1) since lactose stimulated the production of dodecanoic acid, methyl ester, which accounted for 2.55% of the 4.30% of the total ester production in this carbon source. Similarly, the total amount of cyclic alkanes was statistically higher in fungal cultures supplemented with xylose, with cyclohexane isothiocyanate being the prevalent compound (Table 1). Finally, alkenes, ketones and aldehydes were the least well-represented groups of VOCs in terms of number of compounds and their relative percentages (Table 1).

## 4. Discussion

The search for alternative sources of fuels is becoming increasingly important. The metabolism of certain microorganisms is directed toward hydrocarbon production, where fungal endophytes play a predominant role.

After a 7-day incubation period, the fungal biomass was statistically higher in fungal cultures supplemented with glucose, sucrose, and fructose. It was previously reported that these carbon sources are metabolic sugars that promote rapid mycelial growth [26]. On the other hand, xylose and lactose have been reported to be less suitable to support fungal growth [27]. These results are in agreement with previous studies that showed statistically higher growth rates of *F. verticillioides* in solid culture media supplemented with glucose, sucrose, and fructose [4]. Additionally, fructose was reported to stimulate biomass production and the different primary metabolic pathways involved in vegetative growth, such as fatty acid, nucleotide, and amino acid biosynthesis [28].

In the present study, we were able to identify 81 VOCs produced by *F. verticillioides* strain 7600 in submerged cultures supplemented with different carbon sources (glucose, fructose, xylose, sucrose, and lactose). Except for straight-chain alkanes, there were statistically significant differences in the relative percentage of the defined groups of VOCs according to the carbon source provided to the fungus.

To evaluate the diesel potential of the VOCs emitted by *F. verticillioides,* we must consider certain features of diesel fuels that are necessary for satisfactory operation of a diesel engine, such as the cetane number (CN), auto-ignition temperature (AIT), boiling point (BP), and energy content [29] (Table 2).

The CN is an indicative of the fuel ignition characteristics and is related to the time required for the fuel to ignite after injection during compression in a diesel engine. The optimum value of CN is 100, which represents the highest purity of diesel fuel possible. However, in general, values of CN over 45 are considered acceptable and represent suitable ignition delays. This property is related to the fuel composition and varies with hydrocarbon types present in diesel fuels. The relationship between hydrocarbon structural type and CN is generally as follows: alkanes > alkenes > cycloalkanes > aromatics. In the present work, the production of straight-chain alkanes ranged from 22.04% to 24.18% according to the carbon source. Within alkanes, the CN rises with increasing chain length. For example, the CN of octane, nonane, decane, and undecane, four alkanes detected in the present study, are of 64, 72, 77, and 79, respectively (Table 1 and Table 2). Likewise, tetradecane, pentadecane, hexadecane, and octadecane have CN of 96, 98, 100, and 110 respectively [30]. The straight-chain alkanes detected in the present study have potential as diesel fuels because of their high CN values. On the other hand, branched-chain alkanes were highly represented in the analyses, with 30 compounds identified and a total production that ranged from 25.80% to 38.64%, according to the carbon source. Among the molecular features that must be considered when evaluating the CN of a chained-hydrocarbon are the number of branches and the carbon number of the branches, which influence negatively the CN of the compounds [31]. Such is the case of 2-methylpentane, 2,3-dimethylpentane, and 2,2,4-trimethylpentane that have increasingly lower CN values of 34.5, 21.9, and 17.8, respectively [31]. Likewise, 2-methylheptane has a CN value of 52.6 while 2,4-dimethylheptane has a CN of 31 [31,32]. In addition, the ethyl substituted 3-ethyldecane and the propyl substituted 4-propyldecane have CN of 48 and 39, respectively [31,32]. In the present work, most branched-chain alkanes had only one methyl-substitution, while the compounds with more methyl substitutions or with longer side chains were less represented.

Other VOCs, such as alkenes, were poorly represented in the chromatographic analyses; however, tetradecene, which was detected in all fungal cultures, has a CN of 83, i.e., lower than that reported for its saturated counterpart, tetradecane [29]. In addition, even though the production of benzene derivatives (i.e., aromatic hydrocarbons) was significantly higher in glucose and lactose, hydrocarbons with benzene rings tend to have low CN. This pattern is attributable to the fact that the benzylic hydrogen atom is readily extractable, forming a very unreactive benzyl radical which hinders ignition [42]. For example, 1,3-dimethylbenzene, 1,2,3-trimethylbenzene, and 1,2,4,5-tetramethylbenzene have CNs of 7.0, 10.1, and 1.0, respectively [31]. Nevertheless, the CNs of substituted benzenes such as alkyl benzenes increase with increased alkyl chain size [31,43]. For instance, the CN values of two aromatic hydrocarbons detected in the present study, ethylbenzene and propylbenzene, are 6.3 and 16.0, respectively [31]. For these reasons, high aromatic content in distillate fuels lowers the fuel quality while, at the same time, contributing to the formation of soot particulates which are harmful to human health and the environment [29]. On the other hand, certain alkylated cycloalkanes, such as alkylated cyclohexanes, are usually present in diesel fuels [43]. The single-ring cycloalkanes detected in the current work were cyclohexane isothiocyanate, decylcyclopentane, decylcyclohexane, and cyclodecane, which accounted for the 4.47% of the total VOCs produced in xylose-containing medium and lower values for the remaining carbon sources. Similar to alkylated aromatics, the CN of alkylated cyclohexanes increases with increasing alkyl chain size. For example, cyclohexane has a CN of 20.0, while for butylcyclohexane, that figure is 47.8 [31]; therefore, the CN of decylcyclohexane is expected to be even higher than that reported for butylcyclohexane.

The CN of a given VOC is related to its AIT, i.e., the lowest temperature at which ignition occurs by compression [29]. Since diesel engines have no ignition sources (such as a spark or flame), the AIT must be low. In general, the AIT decreases, and thus, the ease with which a molecule reacts increases in the following order: aromatics < branched alkanes < cyclic alkanes < alkanes [33]. For example, the straight-chain alkanes octane, nonane, decane, dodecane, tetradecane, hexadecane, octadecane, and nonadecane have AITs of 220, 206, 208, 204, 202, 202, 235, and 230 °C, respectively [33]. These AIT values are lower compared to those reported for the benzene derivatives ethylbenzene, propylbenzene, and naphthalene with AIT values of 432, 456, and 526 °C, respectively [33,40,41]. Additionally, it has been reported that branching helps to stabilize the molecule, increasing the AIT. This might explain the higher AIT of 1,2,3-trimethylbenzene (470 °C) compared to ethylbenzene and propylbenzene [33]. However, not only the number of branching, but also the position of the ramification affects the AIT. This is the case for 1,2,3-trimethylbenzene and 1,2,4-trimethylbenzene, that showed different AIT values of 470 °C and 515 °C, respectively [33]. Other molecular feature to be considered is the number of double bonds. Molecules that contain these points of unsaturation have reduced susceptibility to autoignition, and thus higher AIT. This can be seen when comparing AIT of tetradecene to that of tetradecane, with values of 235 and 202 °C, respectively [33].

The BP of diesel constituents influences other properties of diesel, such as the AIT and CN values. Most diesel fuels contain hydrocarbons with boiling points within the range of 150 to 380 °C [29]. Compounds with lower BPs would evaporate, accumulate, and mix with air, increasing the risk of pre-ignition [29,44]. Since alkanes are nonpolar molecules, they attract each other by London dispersion forces. The BP of the straight-chain alkanes is higher than that of branched ones with the same molecular weight because the molecular surfaces of the former are more in contact with each other. For example, even though 2,6,10-trimethyldodecane and pentadecane have the same molecular formula (C_15_H_30_), the BP of the former is 248 °C, lower than the BP of the latter, which is 267 °C. The boiling points of the straight-chain alkanes detected in the present study ranged from 126 °C (for octane) to 356 °C (for heneicosane), while the BP of the branched-chain alkanes ranged from 134 °C (for 2,4-dimethylheptane) to 376 °C (for 3-methylheneicosane). Moreover, each additional −CH_2_ group causes an increase of around 20 °C in the BP. For example, decane (C_10_H_22_) has a BP of 174 °C while undecane (C_11_H_24_) has a BP of 195.8 °C [34]. As shown in Table 2, the BP of aromatic hydrocarbons tend to be higher than those of branched- and straight-chain hydrocarbons of similar molecular size. For example, the BP of naphthalene (C_10_H_8_) is 218, rather higher than that of decane (C_10_H_20_). Similar to other nonpolar molecules, aromatic compounds are attracted by London dispersion forces, and even though there are no permanent dipoles on these molecules, the higher BP values are presumably due to the ease with which temporary dipoles can be set up in a system of delocalized electrons.

The energy content (energy density or heating value) is defined as the amount of energy released from the complete combustion of a given mass of a fuel-like compound. Typical energy content values for diesel fuels are around 45.82 MJ/kg. The complete combustion of some of the straight-chain alkanes detected in our study octane, nonane, decane, undecane, tetradecane, pentadecane, hexadecane, octadecane, nonadecane, and eicosane releases between 47 and 48 MJ/kg, while the combustion of the aromatics ethylbenzene and propylbenzene releases 41 MJ/kg [45]. These values are rather superior than those reported for other biofuels, for example, ethanol has an energy content value of only 29 MJ/kg.

The biosynthesis of VOCs by microorganisms proceeds through different metabolic routes [1]. For example, the straight-chain alkanes detected in the present study are typically derived from medium-chain (6–12 carbon atoms) and long-chain (13–22 carbon atoms) fatty acids, which are produced by the condensation of acetyl-CoA and malonyl-CoA units. Further reactions such as oxidation-reduction, decarbonylation, and decarboxylation yield both odd- and even-numbered alkanes, with aldehydes as intermediate precursors [7]. The decarbonylation of the aldehyde precursor results in an alkane that is one carbon shorter than the original fatty acid. This could be the metabolic pathway involved in the biosynthesis of pentadecane and heptadecane detected in the present work, from palmitic acid (16:0) and stearic acid (18:0), two saturated fatty acids previously detected in *F. verticillioides* [46]. Additionally, another pathway for the biosynthesis of long-chain alkanes from fatty acids that involves the formation of 1-alcohols was reported [47]. This pathway could be responsible for the biosynthesis of hexadecane and octadecane by *F. verticillioides* since 1-hexadecanol and 1-octadecanol were also detected in the present work. In fact, both alcohols were detected in higher amounts in fructose- and lactose-containing media. Additionally, palmitic and stearic acids can be modified by different enzymes such as elongases and desaturases. The former adds two carbon atoms to lengthen the carbon chain, producing other long-chain fatty acids; while the latter catalyzes the carbon/carbon double bond formation producing terminal alkenes, such as 1-tetradecene, which was detected in the present study.

In addition to acetyl-CoA and malonyl-CoA, other precursors may also be involved in the hydrocarbon synthetic pathways of *F. verticillioides* since a large number of branched-chain alkanes were produced. Most of them consisted of one and two methyl branches, and the position of the branch was either terminal or in the middle of the molecule. The presence of C4 and C5 branched-chain acyl-CoAs may have been involved in the biosynthesis of terminally branched fatty acids that undergo through further enzymatic reactions to produce different 2-methylakanes [1,48]. This chain reaction could have originated the VOCs 2-methyldecane, 2-methyltridecane, 2-methylpentadecane, 2-methylheptadecane, and 2-methyleicosane in *F. verticillioides*. Still, other metabolic pathways may be responsible for the great diversity of branched-chain alkanes and alcohols produced by *F. verticillioides*. Finally, several benzene derivatives were present in the volatile profile of *F. verticillioides*, especially when glucose and lactose were used as carbon sources. In general, these compounds are synthesized through the shikimate pathway that produces aromatic amino acids, such as phenylalanine, tyrosine, and tryptophan [49]. These results are consistent with previous studies that reported an enhanced aromatic production when using glucose as a sole carbon source. This effect of glucose could be related to the increased carbon flow to the shikimate pathway, stimulating the production or several aromatic intermediates [50,51].

In conclusion, the results presented in the current study show that *F. verticillioides* strain 7600 is able to grow on a variety of low-cost carbon sources and produce many VOCs with diesel potential. Moreover, except for straight-chain alkanes that were synthesized in similar amounts in all carbon sources, the relative percentage of remaining groups of VOCs was increased with specific carbon sources. Further research should include the optimization of other parameters to maximize VOC production, such as nitrogen sources, the pH of the culture medium, and temperature. In addition, monitoring fungal VOCs in other time frames could reveal the production of new VOCs or differences in the amounts of the VOCs that were detected in the present study. Another approach to produce these diesel-like compounds at high yields for commercial exploitation is the genetic manipulation of the metabolic pathways involved in the biosynthesis of the VOCs of interest [48,52,53]. Indeed, another advantage of using *F. verticillioides* strain 7600 as the host organism is the fact that its complete genome sequence has already been characterized.

## Figures and Tables

**Table 1:** Detected VOCs from *F. verticillioides* 7600 grown in GYAM culture medium supplemented with different carbon sources.

KI (C)	KI (T)	VOC	Molecular Formula	Glucose	Fructose	Xylose	Sucrose	Lactose
797	800	Octane	C_8_H_18_	1.88 ± 0.09	0.94 ± 0.21	1.90 ± 0.21	0.75 ± 0.18	0.83 ± 0.18
899	900	Nonane	C_9_H_20_	1.33 ± 0.07	0.30 ± 0.05	0.48 ± 0.03	0.65 ± 0.19	0.68 ± 0.09
999	1000	Decane	C_10_H_22_	3.31 ± 0.14	3.88 ± 0.83	3.24 ± 0.49	3.07 ± 0.91	4.15 ± 0.26
1099	1100	Undecane	C_11_H_24_	5.74 ± 0.14	3.26 ± 0.62	3.41 ± 0.45	4.90 ± 0.94	4.57 ± 0.36
1399	1400	Tetradecane	C_14_H_30_	4.91 ± 0.34	4.63 ± 0.22	5.38 ± 0.17	8.00 ± 0.32	4.80 ± 0.52
1498	1500	Pentadecane	C_15_H_32_	0.82 ± 0.08	1.41 ± 0.19	3.73 ± 0.06	1.15 ± 0.45	0.93 ± 0.10
1598	1600	Hexadecane	C_16_H_34_	2.05 ± 0.05	2.51 ± 0.06	2.28 ± 0.28	1.38 ± 0.27	2.25 ± 0.13
1697	1700	Heptadecane	C_17_H_36_	2.08 ± 0.16	2.85 ± 0.28	1.82 ± 0.14	1.04 ± 0.28	2.44 ± 0.13
1797	1800	Octadecane	C_18_H_38_	1.09 ± 0.10	1.24 ± 0.08	0.60 ± 0.04	0.42 ± 0.12	1.16 ± 0.04
1901	1900	Nonadecane	C_19_H_40_	0.33 ± 0.04	0.48 ± 0.07	0.33 ± 0.14	0.16 ± 0.06	0.43 ± 0.02
1998	2000	Eicosane	C_20_H_42_	0.14 ± 0.02	0.21 ± 0.02	0.07 ± 0.01	0.08 ± 0.03	0.19 ± 0.01
2108	2109	Heneicosane	C_21_H_44_	0.50 ± 0.11	1.75 ± 0.33	0.37 ± 0.05	0.44 ± 0.15	0.98 ± 0.15
*Straight-chain Alkanes*				24.18 ± 0.57 (a)	23.46 ± 1.34 (a)	23.61 ± 0.65 (a)	22.04 ± 1.28 (a)	23.41 ± 1.12 (a)
818	820	2,4-Dimethylheptane	C_9_H_20_	0.52 ± 0.03	0.86 ± 0.11	0.74 ± 0.07	1.09 ± 0.46	0.21 ± 0.03
970	971	3-Methylnonane	C_10_H_22_	3.19 ± 0.15	0.10 ± 0.02	0.14 ± 0.01	0.26 ± 0.05	2.92 ± 0.48
1038	1035	4,5-Dimethylnonane	C_11_H_24_	3.30 ± 0.27	3.78 ± 0.30	2.93 ± 0.16	2.46 ± 0.63	2.44 ± 0.30
1064	1065	2-Methyldecane	C_11_H_24_	1.90 ± 0.14	0.58 ± 0.10	0.61 ± 0.08	0.69 ± 0.15	1.32 ± 0.05
1081	1085	2,4-Dimethyldecane	C_12_H_26_	1.56 ± 0.08	2.21 ± 0.16	1.54 ± 0.07	1.19 ± 0.21	1.10 ± 0.11
1170	1170	3-Methylundecane	C_12_H_26_	0.45 ± 0.03	0.40 ± 0.04	0.43 ± 0.02	0.70 ± 0.09	0.41 ± 0.02
1175	1179	3,6-Dimethylundecane	C_13_H_28_	0.07 ± 0.01	0.15 ± 0.02	0.11 ± 0.01	0.11 ± 0.01	0.07 ± 0.02
1212	1210	2,6-Dimethylundecane	C_13_H_28_	0.69 ± 0.05	1.41 ± 0.11	1.26 ± 0.09	1.66 ± 0.17	0.66 ± 0.14
1269	1271	3-Methyldodecane	C_13_H_28_	1.14 ± 0.07	2.43 ± 0.17	1.90 ± 0.12	2.57 ± 0.28	1.26 ± 0.21
1274	1275	2,6,11-Trimethyldodecane	C_15_H_32_	1.11 ± 0.12	2.21 ± 0.15	1.38 ± 0.10	1.26 ± 0.15	1.27 ± 0.23
1320	1318	2,3,5,8-Tetramethyldecane	C_14_H_30_	0.60 ± 0.04	1.10 ± 0.06	1.18 ± 0.08	1.52 ± 0.14	0.66 ± 0.10
1351	1348	5-Methyltridecane	C_14_H_30_	0.57 ± 0.05	0.63 ± 0.08	2.03 ± 0.12	3.21 ± 0.26	0.39 ± 0.04
1363	1364	2-Methyltridecane	C_14_H_30_	0.72 ± 0.08	1.48 ± 0.12	4.02 ± 0.33	5.98 ± 0.54	0.71 ± 0.10
1374	1368	2,6,10-Trimethyldodecane	C_15_H_32_	0.13 ± 0.01	0.18 ± 0.01	0.32 ± 0.04	0.45 ± 0.04	0.15 ± 0.02
1420	1419	2,6,10-Trimethyltridecane	C_16_H_34_	0.48 ± 0.06	1.23 ± 0.05	1.54 ± 0.08	1.71 ± 0.20	0.68 ± 0.03
1470	1470	3-Methyltetradecane	C_15_H_32_	2.18 ± 0.19	4.96 ± 0.53	6.03 ± 0.47	5.52 ± 1.17	2.14 ± 0.26
1482	1483	6,9-Dimethyltetradecane	C_16_H_34_	0.57 ± 0.04	1.36 ± 0.09	0.60 ± 0.06	0.31 ± 0.04	0.77 ± 0.07
1544	1539	7-Methylpentadecane	C_16_H_34_	1.76 ± 0.14	4.10 ± 0.16	3.25 ± 0.24	2.35 ± 0.55	2.23 ± 0.20
1550	1548	4-Ethyltetradecane	C_16_H_34_	0.15 ± 0.03	0.33 ± 0.10	0.66 ± 0.13	0.61 ± 0.21	0.16 ± 0.02
1562	1563	2-Methylpentadecane	C_16_H_34_	0.85 ± 0.02	1.55 ± 0.18	2.17 ± 0.22	1.64 ± 0.11	0.80 ± 0.04
	1747	2,6,10-Trimethylhexadecane	C_19_H_40_	0.88 ± 0.05	1.75 ± 0.13	0.84 ± 0.05	0.54 ± 0.17	1.24 ± 0.07
1762	1764	2-Methylheptadecane	C_18_H_38_	0.59 ± 0.04	1.07 ± 0.07	0.65 ± 0.07	0.41 ± 0.13	0.82 ± 0.04
1803	1810	2,6,10,14-Tetramethylhexadecane	C_20_H_42_	0.22 ± 0.01	0.45 ± 0.05	0.10 ± 0.01	0.14 ± 0.05	0.44 ± 0.04
1913	1910	2,6-Dimethyloctadecane	C_20_H_42_	0.78 ± 0.09	1.27 ± 0.16	0.40 ± 0.05	0.38 ± 0.13	1.14 ± 0.09
1940	1943	9-Methylnonadecane	C_20_H_42_	0.19 ± 0.03	0.49 ± 0.07	0.15 ± 0.01	0.09 ± 0.03	0.32 ± 0.03
1961	1960	2,6,10,14-Tetramethyloctadecane	C_22_H_46_	0.15 ± 0.02	0.31 ± 0.06	0.07 ± 0.01	0.07 ± 0.02	0.27 ± 0.03
1971	1970	3-Methylnonadecane	C_20_H_42_	0.07 ± 0.01	0.16 ± 0.03	0.04 ± 0.004	0.08 ± 0.03	0.13 ± 0.03
2040	2045	2-Methyleicosane	C_21_H_44_	0.10 ± 0.02	0.25 ± 0.06	0.06 ± 0.004	0.06 ± 0.03	0.20 ± 0.03
2144	2144	10-Methylheneicosane	C_22_H_46_	0.28 ± 0.07	1.01 ± 0.25	0.18 ± 0.02	0.31 ± 0.11	0.48 ± 0.09
2175	2173	3-Methylheneicosane	C_22_H_46_	0.60 ± 0.18	0.83 ± 0.09	0.23 ± 0.03	0.23 ± 0.04	0.56 ± 0.06
*Branched-chain Alkanes*				25.80 ± 0.67 (a)	38.64 ± 0.62 (b)	35.56 ± 0.63 (b)	37.60 ± 1.67 (b)	25.95 ± 1.84 (a)
1238	1231	Cyclohexane isothiocyanate	C_7_H_11_NS	1.06 ± 0.09	1.63 ± 0.17	3.57 ± 0.39	2.32 ± 0.29	1.16 ± 0.21
1552	1550	Decylcyclopentane	C_15_H_30_	0.19 ± 0.03	0.59 ± 0.06	0.27 ± 0.07	0.34 ± 0.06	0.32 ± 0.03
1660	1657	Decylcyclohexane	C_16_H_32_	0.29 ± 0.01	0.29 ± 0.02	0.27 ± 0.04	0.19 ± 0.05	0.47 ± 0.02
		Cyclodecane	C_10_H_20_	0.39 ± 0.04	0.94 ± 0.05	0.36 ± 0.02	0.33 ± 0.09	0.65 ± 0.04
*Cyclic Alkanes*				1.93 ± 0.11 (a)	3.45 ± 0.18 (b)	4.47 ± 0.37 (c)	3.18 ± 0.34 (b)	2.60 ± 0.25 (a)
838	836	2,4-Dimethyl-1-heptene	C_9_H_18_	0.79 ± 0.04	2.78 ± 0.14	1.96 ± 0.19	1.22 ± 0.32	0.51 ± 0.06
1390	1392	1-Tetradecene	C_14_H_28_	0.14 ± 0.01	0.38 ± 0.05	0.56 ± 0.05	0.80 ± 0.05	0.15 ± 0.02
*Alkenes*				0.93 ± 0.04 (a)	3.16 ± 0.16 (c)	2.52 ± 0.17 (b)	2.02 ± 0.29 (b)	0.66 ± 0.09 (a)
857	855	Ethylbenzene	C_8_H_10_	1.60 ± 0.12	0.65 ± 0.16	3.22 ± 0.30	3.77 ± 0.91	1.57 ± 0.24
868	866	1,3-Dimethylbenzene	C_8_H_10_	6.70 ± 0.57	1.13 ± 0.21	5.12 ± 0.32	8.13 ± 1.90	4.48 ± 0.60
916	920	Methoxybenzene	C_7_H_8_O	0.25 ± 0.03	0.20 ± 0.02	0.23 ± 0.03	0.05 ± 0.03	0.30 ± 0.05
953	953	Propylbenzene	C_9_H_12_	0.76 ± 0.03	0.15 ± 0.02	0.20 ± 0.01	0.15 ± 0.03	0.53 ± 0.07
964	961	1,3,5-Trimethylbenzene	C_9_H_12_	6.21 ± 0.12	0.95 ± 0.10	0.95 ± 0.05	0.90 ± 0.20	5.56 ± 0.87
994	997	1,2,3-Trimethylbenzene	C_9_H_12_	16.31 ± 0.61	0.17 ± 0.03	0.37 ± 0.02	0.34 ± 0.08	16.50 ± 3.45
1085	1081	1-Ethyl-2,4-dimethylbenzene	C_10_H_14_	0.74 ± 0.04	0.23 ± 0.02	0.34 ± 0.01	0.30 ± 0.02	0.85 ± 0.02
1122	1117	1,2,4,5-Tetramethylbenzene	C_10_H_14_	0.85 ± 0.02	0.23 ± 0.03	1.23 ± 0.07	3.97 ± 0.52	0.73 ± 0.02
1192	1190	Naphthalene	C_10_H_8_	0.68 ± 0.03	0.48 ± 0.06	0.95 ± 0.05	0.91 ± 0.14	0.49 ± 0.01
1233	1229	Benzothiazole	C_7_H_5_NS	0.69 ± 0.12	0.45 ± 0.04	0.56 ± 0.02	0.37 ± 0.04	0.63 ± 0.07
1250	1247	1,3-Di-tert-butylbenzene	C_14_H_22_	0.30 ± 0.03	1.79 ± 0.03	0.80 ± 0.05	0.66 ± 0.06	0.35 ± 0.03
1330	1331	2-Methylpropyl benzoate	C_11_H_14_O_2_	0.49 ± 0.09	1.05 ± 0.21	2.84 ± 0.56	1.98 ± 0.41	0.34 ± 0.05
*Benzene derivatives*				35.58 ± 0.95 (c)	7.48 ± 0.61 (a)	16.81 ± 0.37 (b)	21.53 ± 3.12 (b)	32.33 ± 3.76 (c)
1878	1880	1-Hexadecanol	C_16_H_34_O	1.00 ± 0.08	1.58 ± 0.15	0.64 ± 0.15	0.67 ± 0.33	1.47 ± 0.04
2072	2070	1-Octadecanol	C_18_H_38_O	0.19 ± 0.02	0.79 ± 0.13	0.23 ± 0.06	0.49 ± 0.25	0.74 ± 0.03
*Straight-chain Alcohols*				1.19 ± 0.10 (a)	2.37 ± 0.20 (b)	0.87 ± 0.20 (a)	1.16 ± 0.57 (a)	2.21 ± 0.05 (b)
1027	1020	2-Ethyl-1-hexanol	C_8_H_18_O	1.79 ± 0.20	2.19 ± 0.18	4.08 ± 0.09	2.18 ± 0.34	2.08 ± 0.08
1227	1229	4,8-Dimethyl-1-nonanol	C_11_H_24_O	0.03 ± 0.002	0.03 ± 0.004	0.09 ± 0.01	0.24 ± 0.03	0.03 ± 0.01
1283	1277	2-Butyl-1-octanol	C_12_H_26_O	0.48 ± 0.03	1.06 ± 0.07	0.97 ± 0.05	0.90 ± 0.07	0.49 ± 0.07
1300	1293	2-Methyl-1-decanol	C_11_H_24_O	0.66 ± 0.04	2.81 ± 0.18	1.95 ± 0.08	1.65 ± 0.07	0.98 ± 0.09
1316	1320	5,9-Dimethyl-1-decanol	C_12_H_26_O	0.51 ± 0.12	1.82 ± 0.12	1.51 ± 0.06	1.40 ± 0.06	0.60 ± 0.06
1487	1492	11-Methyldodecanol	C_13_H_28_O	1.51 ± 0.12	3.19 ± 0.19	0.91 ± 0.12	0.98 ± 0.14	2.02 ± 0.17
1515	1510	2-Hexyl-1-decanol	C_16_H_34_O	0.25 ± 0.01	0.69 ± 0.04	0.73 ± 0.07	0.42 ± 0.08	0.46 ± 0.03
1525	1520	2-Octyl-1-decanol	C_18_H_38_O	0.50 ± 0.05	1.18 ± 0.09	1.45 ± 0.16	1.05 ± 0.25	0.55 ± 0.07
1995	1989	2-Hexyl-1-dodecanol	C_18_H_38_O	0.75 ± 0.14	1.01 ± 0.15	0.44 ± 0.06	0.22 ± 0.03	0.85 ± 0.10
2054	2060	9-Octadecen-1-ol	C_18_H_36_O	0.16 ± 0.03	2.21 ± 0.21	0.74 ± 0.24	1.23 ± 0.62	0.22 ± 0.03
2188	2188	2-Octyl-1-dodecanol	C_20_H_42_O	0.18 ± 0.04	0.52 ± 0.05	0.22 ± 0.03	0.17 ± 0.06	0.26 ± 0.04
*Branched-chain Alcohols*				6.82 ± 0.25 (a)	16.71 ± 0.39 (e)	13.09 ± 0.20 (d)	10.44 ± 0.89 (c)	8.54 ± 0.59 (b)
1473	1475	Decanoic acid, 2-propenyl ester	C_13_H_24_O_2_	0.51 ± 0.03	1.39 ± 0.03	1.09 ± 0.07	0.89 ± 0.15	0.68 ± 0.03
1887	1886	Pentadecanoic acid, 14-methyl-, methyl ester	C_17_H_34_O_2_	0.17 ± 0.02	0.35 ± 0.04	0.12 ± 0.02	0.09 ± 0.03	0.25 ± 0.01
1918	1914	Pentadecanoic acid, isopropyl ester	C_18_H_36_O_2_	0.26 ± 0.03	0.88 ± 0.03	0.15 ± 0.02	0.13 ± 0.04	0.34 ± 0.03
-	2382	Hexanedioic acid, bis (2-ethylhexyl) ester	C_22_H_42_O_4_	0.45 ± 0.06	0.58 ± 0.13	0.18 ± 0.04	0.12 ± 0.03	0.48 ± 0.17
1605	1606	Dodecanoic acid, methyl ester	C_13_H_26_O_2_	2.10 ± 0.14	1.32 ± 0.10	1.39 ± 0.24	0.77 ± 0.25	2.55 ± 0.15
*Esters*				3.49 ± 0.20 (b)	4.52 ± 0.21 (c)	2.93 ± 0.20 (b)	2.00 ± 0.47 (a)	4.30 ± 0.17 (c)
982	983	3-Octanone	C_8_H_16_O	-	0.08 ± 0.004	0.08 ± 0.01	-	-
1204	1204	Decanal	C_10_H_20_O	0.08 ± 0.01	0.07 ± 0.01	0.08 ± 0.01	0.06 ± 0.003	0.05 ± 0.01
1815	1816	Hexadecanal	C_16_H_32_O	0.13 ± 0.04	0.12 ± 0.01	0.09 ± 0.01	0.04 ± 0.01	0.08 ± 0.01
*Ketones and Aldehydes*				0.21 ± 0.04 (b)	0.27 ± 0.01 (b)	0.25 ± 0.02 (b)	0.10 ± 0.01 (a)	0.13 ± 0.01 (a)

KI (C): calculated Kovats Retention Index; KI (T): theoretical Kovats Retention Index. VOCs are expressed in relative percentage (%) by peak area normalization. Total percentage of each group of VOCs are expressed as means ± SE. Within each group of VOCs, values with different letters indicate statistical differences among carbon sources according to the DGC test (*p* < 0.05).

**Table 2:** Cetane number (CN), autoignition temperature (AIT), and boiling point (BP) values of some VOCs detected and discussed in the current study.

VOC	CN *	AIT (°C) *	BP (°C) *	Reference
Octane	64.0	220	126	[30,33,34]
Nonane	72.0	206	151	[30,33,34]
Decane	77.0	208	174	[30,33,34]
Undecane	79.0	198	196	[30,33,34]
Dodecane	80.0	204	216	[30,33,34]
Tetradecane	96.0	202	253	[30,33,34]
Pentadecane	98.0	195	267	[30,34,35]
Hexadecane	100.0	202	287	[30,33,34]
Heptadecane	105.0	203	302	[30,34,36]
Octadecane	110.0	235	317	[30,33,34]
Nonadecane	110.0	230	330	[30,33,34]
Eicosane	110.0	232	343	[30,34,36]
Heneicosane	N/A	220	356	[34,36]
2-Methylpentane	34.5	303	60	[31,33,34]
2,3-Dimethylpentane	21.9	337	90	[31,33,34]
2,2,4-Trimethylpentane	17.8	418	99	[31,33,34]
3-Ethyldecane	48.0	N/A	209	[31,34]
4-Propyldecane	39.0	N/A	236	[31,34]
2-Methylheptane	52.6	N/A	116	[31,34]
2,4-Dimethylheptane	31.0	N/A	134	[32,34]
3-Methylnonane	56.0	N/A	168	[34,37]
3-Methylundecane	69.0	N/A	210	[34,37]
2,6-Dimethylundecane	50.0	N/A	218	[34,37]
2,6,10-Trimethyldodecane	59.1	N/A	248	[34,38]
4-Ethyltetradecane	73.0	N/A	282	[34,37]
2-methylpentadecane	100.0	N/A	282	[34,37]
2-Methylheptadecane	101.0	N/A	311	[34,37]
3-Methylheneicosane	N/A	N/A	376	[34]
1-Tetradecene	83	235	247	[29,33,34]
Ethylbenzene	6.3	432	136	[31,33,34]
1,3-Dimethylbenzene	7.0	527	139	[31,34,39]
Propylbenzene	16.0	456	159	[31,33,34]
Naphthalene	1.0	526	218	[34,40,41]
1,2,3-Trimethylbenzene	10.1	470	176	[31,33,34]
1,2,4-Trimethylbenzene	8.9	515	169	[31,33,34]
1,2,4,5-Tetramethylbenzene	1.0	N/A	194	[31,34]
Cyclohexane	20.0	260	80.7	[31,33,34]
Butylcyclohexane	47.8	246	181	[31,33,34]
1-Hexadecanol	68.0	N/A	249	[30,34]
1-Octadecanol	81.0	177	210	[30,34]
2-Ethyl-1-hexanol	23.4	288	184	[30,33,34]
3-Octanona	35.2	N/A	167	[30,34]

* CN, AIT, and BP values were obtained from references listed in the bibliography.

## Data Availability

Not applicable.
